# Supplementary material for: Proteomic Stratification of Prognosis and Treatment Options for Small Cell Lung Cancer
Source: Genomics Proteomics Bioinformatics. 2024 Apr 18;22(2):qzae033. doi: 10.1093/gpbjnl/qzae033 (PMC11423856; doi:10.1093/gpbjnl/qzae033)
Supplement: qzae033_Supplementary_Data [file qzae033_supplementary_data.zip › Supplementary material captions.docx]

Supplementary material

Figure S1 Univariate prognostic analysis of common clinical pathological factors

Figure S2 Representative IHC staining and distribution of common neuroendocrine biomarkers in SCLC

A. Representative IHC staining of ASCL1, NeuroD1, and YAP1. B. The expression of ASCL1, NeuroD1, and YAP1 was not distributed differently across the three subtypes. IHC, immunohistochemistry.

Figure S3 SCLC proteomic subtype was an independent prognostic factor confirmed by multivariate Cox analysis

Table S1 Clinical pathological data of discovery cohort

Table S2 Proteomic results (FOT) of each case in the discovery cohort

Table S3 Proteins for the non-negative matrix factorization consensus clustering

Table S4 Fifty-eight signature proteins for the predictive classifier model

Table S5 Clinical pathological data of validation cohort

Table S6 Proteomic results (FOT) of each case in the validation cohort

Table S7 Signature proteins for each subtype

Table S8 Clinical pathological data of the immunotherapy cohort

Table S9 Proteomic results (FOT) of each case in the immunotherapy cohort
