# Supplementary material for: Proteomic Stratification of Prognosis and Treatment Options for Small Cell Lung Cancer
Source: Genomics Proteomics Bioinformatics. 2024 Apr 18;22(2):qzae033. doi: 10.1093/gpbjnl/qzae033 (PMC11423856; doi:10.1093/gpbjnl/qzae033)
Supplement: qzae033_Supplementary_Data [file qzae033_supplementary_data.zip › SF1.pdf]

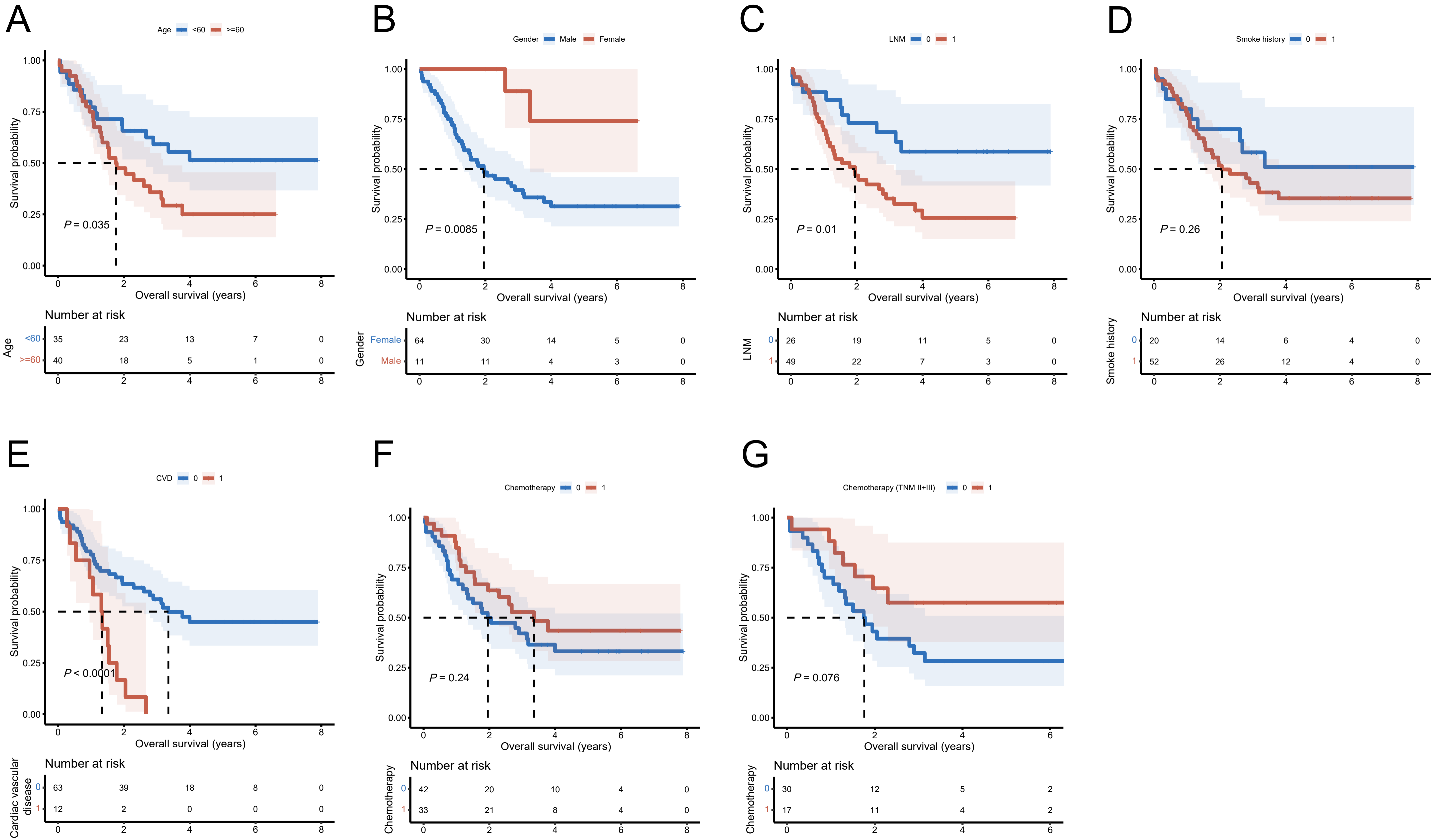
A
Age <60 >=60
Survival probability
1.00 0.75 0.50 0.25 0.00
P = 0.035
Overall survival (years)
0 2 4 6 8
Number at risk
Age
<60 35 23 13 7 0
>=60 40 18 5 1 0
B
Gender Male Female
P = 0.0085
Number at risk
Gender
Female 64 30 14 5 0
Male 11 11 4 3 0
C
LNM 0 1
P = 0.01
Number at risk
LNM
0 26 19 11 5 0
1 49 22 7 3 0
D
Smoke history 0 1
P = 0.26
Number at risk
Smoke history
0 20 14 6 4 0
1 52 26 12 4 0
E
CVD 0 1
P < 0.0001
Number at risk
Cardiac vascular disease
0 63 39 18 8 0
1 12 2 0 0 0
F
Chemotherapy 0 1
P = 0.24
Number at risk
Chemotherapy
0 42 20 10 4 0
1 33 21 8 4 0
G
Chemotherapy (TNM II+III) 0 1
P = 0.076
Overall survival (years)
0 2 4 6
Number at risk
Chemotherapy
0 30 12 5 2
1 17 11 4 2
