# Supplementary figures and images for: Proteomic Stratification of Prognosis and Treatment Options for Small Cell Lung Cancer
Source: Genomics Proteomics Bioinformatics. 2024 Apr 18;22(2):qzae033. doi: 10.1093/gpbjnl/qzae033 (PMC11423856; doi:10.1093/gpbjnl/qzae033)

A

Positive

Negative

ASCL1

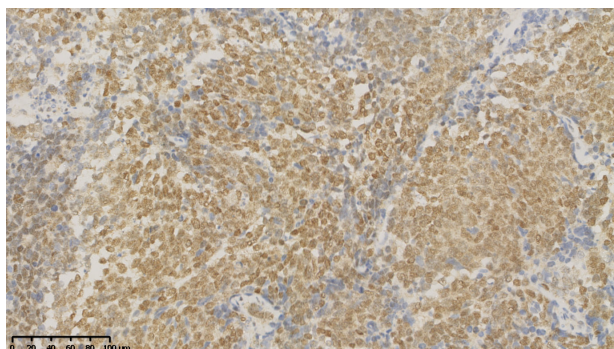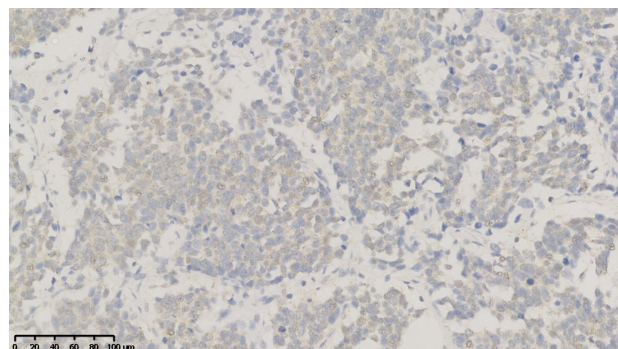

NeuroD1

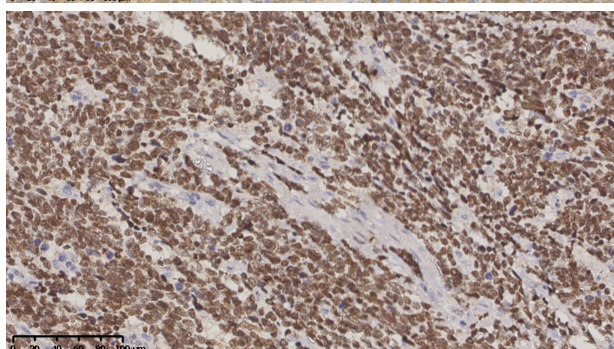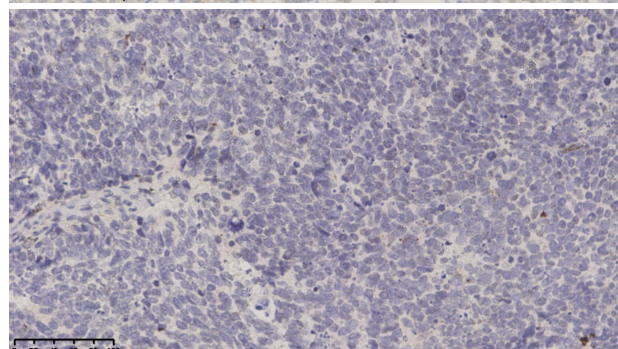

YAP1

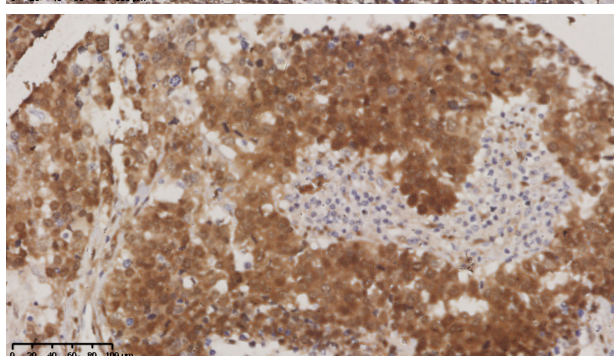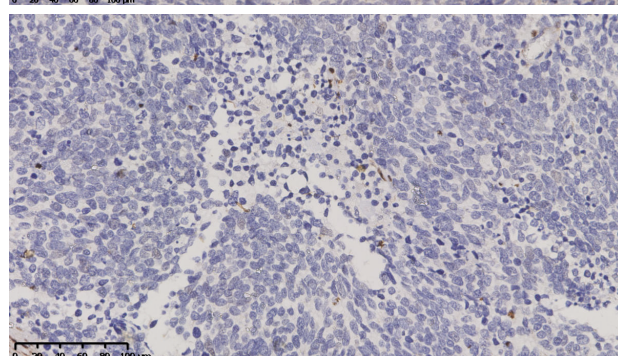

B

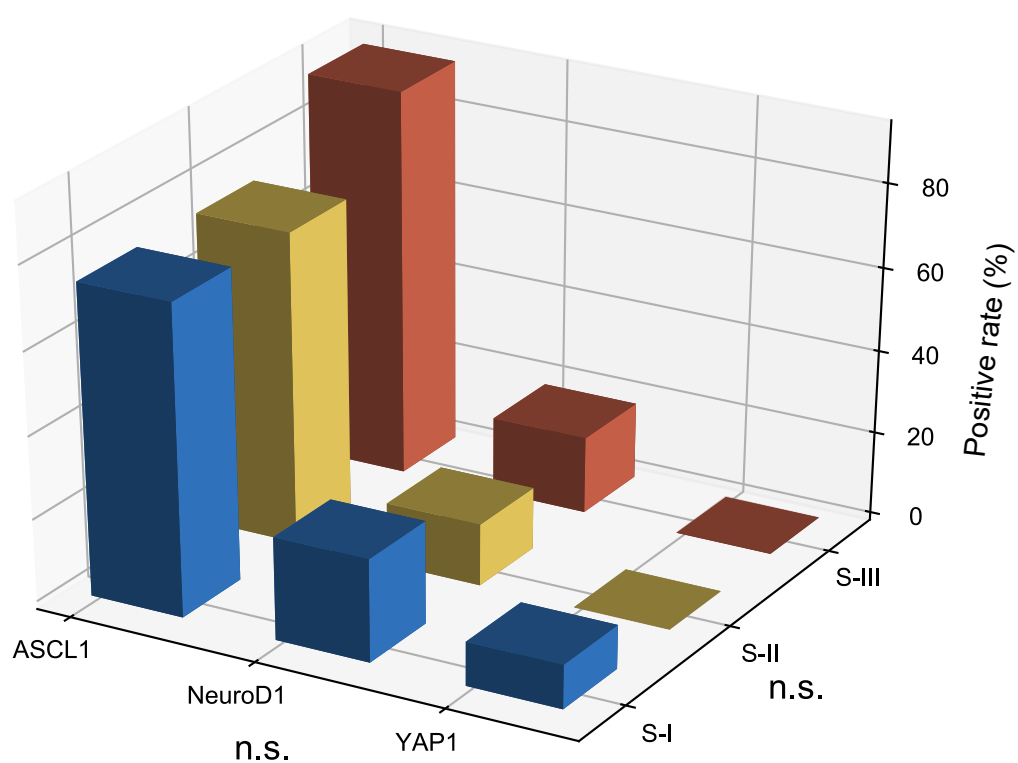

Supplement: qzae033_Supplementary_Data [file qzae033_supplementary_data.zip › SF2.pdf]
